# Harnessing Minimal Residual Disease as a Predictor for Colorectal Cancer: Promising Horizons Amidst Challenges

**DOI:** 10.3390/medicina59101886

**Published:** 2023-10-23

**Authors:** Xiaofen Wen, Donatella Coradduzza, Jiaxin Shen, Antonio Mario Scanu, Maria Rosaria Muroni, Matteo Massidda, Vincenzo Rallo, Ciriaco Carru, Andrea Angius, Maria Rosaria De Miglio

**Affiliations:** 1 Department of Biomedical Sciences, University of Sassari, 07100 Sassari, Italy; x.wen@studenti.uniss.it (X.W.); dcoradduzza@uniss.it (D.C.); j.shen@studenti.uniss.it (J.S.); carru@uniss.it (C.C.); 2 Department of Medical Oncology, Cancer Hospital of Shantou University Medical College, Shantou 515041, China; 3 Department of Hematology, The First Affiliated Hospital of Shantou University Medical College, Shantou 515041, China; 4 Department of Medicine, Surgery and Pharmacy, University of Sassari, 07100 Sassari, Italy; scanu@uniss.it (A.M.S.); mrmuroni@uniss.it (M.R.M.); mmassidda@uniss.it (M.M.); 5 Istituto di Ricerca Genetica e Biomedica (IRGB), Consiglio Nazionale delle Ricerche, CNR, Cittadella Universitaria di Cagliari, Monserrato, 09042 Cagliari, Italy; vincenzo.rallo@irgb.cnr.it (V.R.); andrea.angius@irgb.cnr.it (A.A.)

**Keywords:** colorectal cancer (CRC), Minimal Residual Disease (MRD), MRD techniques, MRD clinical application in CRC

## Abstract

Minimal Residual Disease (MRD) detection has emerged as an independent factor in clinical and pathological cancer assessment offering a highly effective method for predicting recurrence in colorectal cancer (CRC). The ongoing research initiatives such as the DYNAMIC and CIRCULATE-Japan studies, have revealed the potential of MRD detection based on circulating tumor DNA (ctDNA) to revolutionize management for CRC patients. MRD detection represents an opportunity for risk stratification, treatment guidance, and early relapse monitoring. Here we overviewed the evolving landscape of MRD technology and its promising applications through the most up-to-date research and reviews, underscoring the transformative potential of this approach. Our primary focus is to provide a point-to-point perspective and address key challenges relating to the adoption of ctDNA-based MRD detection in the clinical setting. By identifying critical areas of interest and hurdles surrounding clinical significance, detection criteria, and potential applications of basic research, this article offers insights into the advancements needed to evaluate the role of ctDNA in CRC MRD detection, contributing to favorable clinical options and improved outcomes in the management of CRC.

## 1. Introduction

Colorectal cancer (CRC) ranks as the third most common malignancy globally and the second leading cause of cancer-related deaths [1,2]. For early to mid-stage colorectal cancer patients, the mainstay of treatment is surgical resection, where high-risk individuals are followed by postoperative adjuvant chemotherapy (ACT). The foremost challenge lies in the identification of high-risk individuals to tailor effective adjuvant therapy [3]. Likewise, significant is the timely detection of tumor recurrence, warranting prompt intervention. Currently, apart from the microsatellite instability (MSI) marker, clinical decision for ACT in CRC patients is predominantly based on traditional clinical and pathological factors. Nevertheless, risk stratification inferred based on these factors might not yield the desired accuracy, leading to either excessive or insufficient treatments [4].

Minimal Residual Disease (MRD) refers to tiny cancerous lesions that persist in the body following surgical resection or other curative treatments. These tiny remnants become targets with high difficulty of detection by conventional morphological methods and represent a hidden stage of tumor progression. The MRD concept was mainly applied in hematologic malignancies. The clinical assessment of residual solid tumors is steadily advancing from macroscopic evaluations using imaging techniques [such as X-ray, computed tomography (CT), and ultrasound] to cellular and molecular levels utilizing technologies that can detect circulating tumor cells (CTCs) and circulating tumor DNA (ctDNA). In clinical practices, this advancement will enable the refinement of residual tumor assessment criteria: the MRD definition will evolve as technology advances and the understanding of tumors in human healthcare evolves [5,6].

Classical imaging assessment for CRCs are non-invasive but require a certain tumor size and high imaging resolution. CT and PET-CT scans for dynamic detection introduce higher radiation exposure, resulting in the need for time intervals between evaluations. Serological tests, including typical tumor markers carcinoembryonic antigen (CEA) and CA19-9, are another frequently employed method. Unfortunately, evidence of CEA showed more sensitivity to advanced CRC compared to early diseases [7], while a drawback of using an increase in CA19-9 is due to its non-specificity and shared sensitivity with non-cancerous conditions like poorly controlled diabetes mellitus [8]. These limits to application do not provide absolute diagnostic precision. Furthermore, the established diagnostic gold standard is strictly dependent on pathological biopsy specimens. The sample collection process presents some critical issues, particularly when biopsy specimens are obtained from polyps during colonoscopy or from suspected metastatic lesions [9]. The feasibility of invasive procedures for continuous pathological biopsy is limited, so such assessments are typically conducted only at critical time points [10,11].

At present, liquid biopsy techniques emerge as one of the most effective approaches for assessing MRD. Specifically, ctDNA consists of the fragments of tumor cell-released DNA that hold tumor-specific information. These fragments are part of the cell-free DNA (cfDNA) present in the plasma [12]. By analyzing blood samples, it becomes possible to identify tumor-specific DNA, enabling precise evaluation of residual tumors in patients. Unlike traditional imaging and pathological tests, ctDNA exhibits higher sensitivity and specificity, making it a valuable tool for dynamically monitoring tumor burden in patients [13,14]. Moreover, ctDNA-MRD monitoring improves the capability to detect tumor residuals or recurrences 8–12 months earlier compared to conventional approaches [15].

Numerous articles and published studies support the usefulness of ctDNA for MRD assessment in CRC patients. Concurrently, ongoing ctDNA-guided adjuvant clinical trials are underway to define prospective adjuvant therapy strategies for CRC patients. This review article aims to introduce a novel perspective, focusing on targeted application and the real-world challenges in using MRD assessment within the CRC context. Five aspects of pressing issues to be discovered have been and comprehensively discussed, aiming to provide both theory and practical evidence to facilitate the profound progress of MRD in terms of clinical application significance, detection technology standards, and increase in scientific research in CRC. We want to explore alternative viewpoints and propose the current key areas of interest and difficulties regarding the utilization of ctDNA in the real-world clinical environment for MRD detection and meaning in CRC. A comprehensive search strategy was devised and employed for the literature databases PubMed, Scopus, and Web of Science between January 2010 and September 2023. The keywords “Colorectal cancer”, “Minimal Residual Disease”, “MRD techniques”, “Guideline of CRC”, “Recurrence of CRC”, “Metastatic CRC” were used to refine the scope of the literature identified in the initial searches. Boolean operators, such as AND/OR, were strategically employed to amalgamate the search terms effectively. Combinations of these terms were used to screen the mentioned databases for relevant content: title, abstract, and full content, respectively. Only studies written in English were considered for evaluation. Next, all reference lists of identified papers were examined and then a hand search for identified relevant studies was conducted. Pre-screening and screening selection removed duplicate studies, foreign language studies, irrelevant studies, and studies for which updated research was unavailable.

## 2. Applying MRD Technique: When Is It suitable?

At the moment, various authoritative sources, including the National Cancer Institute (NCI) [16], the National Comprehensive Cancer Network (NCCN) [17], and the Chinese Society of Clinical Oncology (CSCO) guidelines [18], have recognized the valuable applications of ctDNA testing. These include (1) assessing preoperative risks, stratifying risks, and predicting patient prognosis in both pre- and post-operative settings; (2) guiding decisions on adjuvant therapy and its adjustment (e.g., determining the need for chemotherapy and selecting appropriate treatment combinations); and (3) enabling regular follow-up and dynamic monitoring after radical therapy, facilitating early intervention in case of recurrence. However, despite such evidence, it is of note that both NCCN and European Society for Medical Oncology (ESMO) [19] guidelines currently do not consider application of ctDNA testing as routine recommendation.

It is crucial to prioritize the practical effectiveness of MRD during the entire CRC health care process. Sensitivity holds a particular significance in clinical evaluations. During early screening, tumor-related DNA content in the blood might be extremely low (less than 0.01%) [19,20]. Technologies like ctDNA or liquid biopsy necessitate remarkably high sensitivity. The first aspect of understanding high-sensitivity MRD is to compare two scenarios: patients with pre-diagnosed CRC and patients with metastatic CRC (mCRC), where tumor-related DNA content in the blood is notably higher. In these instances, high sensitivity in MRD testing methods is not critical. Traditional and established testing methods (imaging, serological markers, histopathological findings from surgical specimens) can also serve their purpose. This highlights that MRD testing involves gradually deepening levels of sensitivity, thus shaping our initial comprehension.

Furthermore, the discussion revolves around the clinical scenarios in which the application of MRD proves necessary and pertinent. Typically, in the context of a “curative” therapeutic approach and complete remission (CR) confirmed through routine diagnostic means, the MRD is considered when the need arises to validate the true or deeper extent of the achieved CR. Such instances might include (1) postoperative assessment after curative resection for stages I-III CRC [21], (2) evaluation of post-curative resection (R0) for stage IV CRC [22,23], (3) locally advanced CRC achieving clinical CR after neoadjuvant therapy (NAT) [24], (4) MSI-H CRC showing no tumor activity after immunotherapy [25], and (5) clinical judgment of CR in mCRC patients after systemic treatment. We will discuss another consideration: how ctDNA can assist in identifying treatment targets in advanced stage mCRC patients.

## 3. Personalized Treatment Choices: Can MRD Inform Adjuvant Decisions?

Patients undergoing postoperative ACT primarily aim to eliminate micro-metastatic disease, which we consider as MRD. Clinical practice generally categorizes stage II CRC patients into low, intermediate, and high-risk groups based on clinical pathology parameters. Specifically, low-risk stage II patients avoid chemotherapy, intermediate-risk stage II patients consider monotherapy, and high-risk stage II patients must receive chemotherapy combined with Oxaliplatin [26]. For a long time, factors contributing to the designation of high-risk stage II CRC included conditions like obstruction, perforation, or vascular invasion, often combined with tumoral deficiency of DNA mismatch repair (MMR) enzyme status. Several meta-analyses have evaluated that the benefit of postoperative ACT for stage II CRC patients is approximately no greater than 5%, necessitating a more refined and optimal approach to identify the subgroup that might benefit from adjuvant therapy [27,28,29,30].

Undeniably, the transition from detectable to undetectable ctDNA after ACT could signify treatment effectiveness. This might carry two noteworthy implications in future clinical practice. Firstly, MRD results could substantially contribute to personalized adjuvant treatment decisions. Patients traditionally categorized as stage I, and thus commonly excluded from chemotherapy regimens, have been observed to have a relapse rate of 2.3% to 4.7% within 5 years after curative resection [31,32]. MRD assessment might effectively identify high-risk patients who previously presented challenges to categorize, offering significant guidance in treatment selection and strategies. Secondly, it might enable precise selection of patients for chemotherapy. Despite chemotherapy being a common clinical approach for CRC, its actual benefit is for a limited patient subset [33,34]. MRD assessment could accurately identify which patients will benefit from chemotherapy, thereby optimizing the balance between treatment efficacy and potential risks.

The results of groundbreaking CIRCULATE-Japan study [35] for postoperative stage II-IV colon cancer (GALAXY) confirm that the patients’ group of MRD positivity at four weeks after surgery benefit significantly from ACT, at all stages of CRC. Conversely, high-risk stage II and III patients with MRD negativity at four weeks postoperatively do not gain significant benefits from ACT (*p* = 0.63). ctDNA during ACT dynamically predicts therapeutic benefits, showing considerably better outcomes for ctDNA-negative patients compared to ctDNA-positive patients. The Australian DYNAMIC study [36] is another milestone, exploring whether ctDNA-guided patients can reduce ACT without increasing the risk of relapse. The proportion of the trial group receiving ACT was lower than the control group (15.3% vs. 27.9%, OR = 2.14, *p* = 0.002), but the rates of two-year and three-year disease-free patients were comparable (2-year disease-free survival rates of 93.5% vs. 92.4%, 3-year disease-free survival rates of 91.7% vs. 92.4%, HR 0.96; 95% CI, 0.51–1.82). These findings indicate that ctDNA-guided ACT can avoid ineffective chemotherapy while still ensuring curative effects.

The DYNAMIC-III study approach involves initial MRD testing of patients with stage III colon cancer. If the result is negative, a deescalated treatment strategy is pursued, which consist of transitioning from two-drug therapy to combination therapy, switching from combination therapy to single-agent treatment, or changing the treatment duration from 6 months to 3 months, or even temporarily stopping treatment. These studies are ongoing, and we are currently awaiting the results of this approach. Interestingly, the IDEA study conducted in France [37] revealed that not all patients with CRC stage III need conventional 6-month standard ACT after surgery. In these cases, the addition of MRD testing further refines postoperative ACT. It helps clinicians identify which patients need a 6-month course, which may benefit from a 3-month course, and which cases might derive advantages from an extended ACT duration. This refinement is in line with our aspirations in postoperative ACT strategies.

It must be recognized that MRD testing cannot exclusively dictate treatment decisions. Current studies progressively agree on the critical importance of the first postoperative MRD assessment. Another crucial timepoint is three-month postoperatively: if the MRD test gives a positive result, chemotherapy can be contemplated; conversely, if the MRD status is negative, chemotherapy can be stopped. Nonetheless, challenges persist. For example, in cases where a patient undergoing all ACT tests positive for MRD during routine follow-up, implementation of further intervention could be impractical. Additionally, informing patients about potential recurrence risks postoperatively can raise ethical concerns [38]. Extensive large-scale clinical research remains imperative to determine whether therapeutic intervention based on MRD positivity can reverse patients’ prognosis. Table 1 summarizes more representative publications for clinical relevance of MRD at different stages of CRC.

## 4. Optimizing CRC Risk Classification with MRD: How Does It Match Patient Prognosis?

The ultimate goal of CRC treatment and surveillance across all stages is to identify and prevent recurrence as early as possible after curative treatment. Complete remission, defined as the absence of cancer signs based on imaging and laboratory tests, indicates treatment effectiveness. However, the introduction of the MRD concept allows a molecular-level exploration of CR within clinical application. Some encouraging research results indicate that ctDNA could serve as an important predictive biomarker for MRD, potentially addressing the challenge of accurate risk stratification for postoperative CRC patients.

A ctDNA-based MRD test can detect CRC recurrence earlier than imaging and traditional clinical methods, providing a valuable opportunity for early intervention. Updated data from the large observational GALAXY study [47] indicated that in CRC patients with stage II to IV, regardless of BRAF V600E or MSI status, MRD positivity four weeks after intervention is the strongest prognostic risk factor for disease-free survival (DFS). Robust evidence from the FUSCC data [48] has supported a connection between the ctDNA status and the risk of CRC recurrence. ctDNA has been shown to provide early predictions of CRC recurrence risk at any assessed time point (either one week before surgery or one week after surgery). The combination of ctDNA and CEA is able to slightly improve the efficacy of recurrence detection (AUC = 0.849). Nevertheless, the disparity compared with the individual ctDNA testing (AUC = 0.839) does not exhibit statistical significance.

Other observational studies [42,49] and ongoing prospective research [50] have also confirmed the prognostic value of ctDNA assessment in postoperative patients with different stages of CRC. Collectively, these significant studies suggest that clinical staging combined with risk factors is currently the main basis for risk stratification in CRC patients. Hence, the integration and application of ctDNA with clinical features hold the promise to further refine risk stratification, facilitating better prediction of recurrence.

## 5. Navigating the Pitfalls: What Challenges Emerge from Varying MRD Techniques?

The proliferation of MRD studies has generally yielded positive research conclusions, but significant disparities persist among concerning MRD detection methods and interpretation criteria. MRD detection based on Next-Generation Sequencing (NGS) is predominantly divided into two prominent streams: Tumor-informed and Tumor-naïve [21]. These approaches exhibit competitive attributes in terms of sensitivity and specificity due to reliance or independence on primary tumor tissue, use of custom or fixed panels, and variation in assessment periods. Deeper consideration deserves the “Tumor-informed” assay as it might neglect the spatial and temporal heterogeneity inherent in cases where primary lesions and metastatic sites might diverge concurrently [51]. The REVERCE study indicates that despite cetuximab treatment, CRC patients maintain a substantial likelihood of acquiring secondary mutations, creating a challenge in identifying tumor heterogeneity when creating gene panels [52]. Conversely, the adoption of fixed-panel assays fails to sufficiently customize information, placing higher requirements on the sensitivity of detection technology.

DNA methylation stands as a significant indicator of the malignancy of tumor cells. Detection of methylation allows determination by analysis of genes in the blood to detect the presence of tumors. Recognized as an innovative marker for MRD, DNA methylation has the advantage of not requiring complete sequencing of the tumor tissue genome. It can be used directly for blood testing, avoiding the risk of a false-positive from mutations originating in normal tissue, non-malignant conditions, or clonal hematopoiesis [53]. Research conducted in both France and China has presented findings on a two-gene and multi-gene methylation marker detection method for CRC blood samples, providing guidance for determining appropriate ACT strategies [37,48]. Advancement in methylation technology will progressively integrate into MRD assessment, reducing economic and time costs.

The specificity of ctDNA is another crucial consideration in the development of all MRD techniques. The purpose of ctDNA technology application is to guide subsequent clinical interventions and treatments. Low specificity in detection could significantly impact treatment choices, leading to false negatives and false positives. Recently, driver genes have revealed that previously mutated RAS genes, after undergoing systemic treatments like ACT and anti-angiogenic targeted therapy, can revert to a wildtype state, known as NeoRAS [54]. This alteration allows targeted treatment with EGFR monoclonal antibodies. Rapid advance of ctDNA technology provides a technical basis for identifying and characterizing the NeoRAS phenomenon. In the future, the concept of MRD should be integrated into NeoRAS detection techniques, which is expected to further enhance the effectiveness of real-time analysis of targeted therapy resistance.

Lastly, establishing the optimal timing for reliable MRD detection through blood collection after surgery or ACT remains uncertain. The most compelling evidence highlights three distinct crucial timeframes: within the initial 2 weeks to 1 month following surgery, determining the necessity and approach for adjuvant chemotherapy [44,55]; the first 3-month mark of adjuvant chemotherapy, making decisions regarding subsequent treatments [46]; and continual follow-ups of MRD after systemic therapy, in case of a significant higher recurrency associated with a positive MRD status [38]. Interestingly, a large-scale retrospective study used a tumor-informed detection method to analyze the correlation between postoperative cfDNA dynamics and ctDNA positivity, aiming to explore the best timing for postoperative ctDNA detection [56]. cfDNA concentration significantly increased during the first 0–2 weeks after surgery and during ACT, potentially due to cell death and cfDNA shedding (*p* < 0.0001), followed by a gradual decrease in cfDNA concentration over the subsequent 2–8 weeks. Notably, plasma cfDNA levels did not significantly affect ctDNA detection rates within various MRD windows. This finding impacts the practical application of personalized ctDNA testing, allowing earlier windows (two-week after surgery) and potentially influencing the design of clinical trials using ctDNA as a pivotal biomarker.

## 6. More for mCRC: Is There an Expectation Gap?

Among new colorectal cancer diagnoses, 20% of patients have metastatic disease at onset and another 30–50% go on to liver or lung metastases after curative resection [57,58]. For these patients who undergo transformative therapies and surgical resection of the primary and metastatic lesions, assessment of recurrence risk requires not only conventional clinical indicators but also the potential involvement of MRD. The underlying mechanism stems from the fact that metastatic tumors can access other organs and tissues via lymphatic or blood vessels, subsequently releasing ctDNA at these sites [59]. The location of metastatic lesions also influences ctDNA concentration and detection rates. Recent studies suggest that in mCRC with single-organ metastases, the concentration of ctDNA is significantly lower in cases of lung-only or peritoneal-only metastases compared to liver metastases [60].

Increased ctDNA concentration is generally associated with poorer prognosis in the majority of mCRC patients. For instance, in RAS wild-type mCRC cases, a significant correlation progression-free survival (PFS) between higher pretreatment levels of ctDNA and worse PFS and shorter overall survival (OS) compared to cases with lower pretreatment ctDNA levels has been demonstrated [61]. However, there is currently no standardized definition for the threshold of high-level ctDNA, and there is still no consensus on how this finding should guide the selection of treatment strategies. It was also noted that levels of CEA and baseline lesion measurements from imaging were not indicative of prognosis. This is in line with earlier findings that propose CEA, despite being accessible and convenient, as an unreliable indicator of treatment response in mCRC. In many patients, CEA levels may not initially rise, but they might increase in patients in response to chemotherapy [62,63]. Therefore, exploring the potential of dynamic MRD detection to improve this situation remains a promising area for ongoing investigation.

The potential utility of ctDNA-based MRD detection in evaluating the treatment response of mCRC remains an area yet to be fully explored. It has been observed that some mCRC patients do not respond well to or benefit from standard-of-care treatments such as NAT [64] or maintenance chemotherapy. In the neoadjuvant setting, ctDNA has shown promise as a precise predictor of treatment response. It has the potential to anticipate the effectiveness of NAT as early as before the second cycle, optimizing the treatment strategy for mCRC patients [65]. Additionally, the potential of ctDNA to aid in maintenance chemotherapy for stable mCRC is supported by research indicating that reduced ctDNA levels after chemotherapy are linked to better clinical outcomes in mCRC, although the ideal timing for ctDNA assessment post-chemotherapy remains uncertain [66,67,68]. If MRD assessment later proved to inform decision-making, such as extending first-line FLOFOX or FLOFIRI therapy or temporary interruption of therapy, it has the potential to minimize the exposure of stable mCRC patients to the adverse effects of chemotherapy [69]. Furthermore, advancements in ctDNA-guided translational research have led the identification of new therapeutic targets, improving the treatment outcome of patients harboring BRAF V600E mutation [70], ErBB2 alterations [71], NTRK gene fusions [72] and KRAS(G12C) mutation [73]. Currently there are no approved or recommended immunotherapy regimens or combination therapies for microsatellite stable/MMR proficient mCRC [74].

## 7. Conclusions

**Figure 1:**
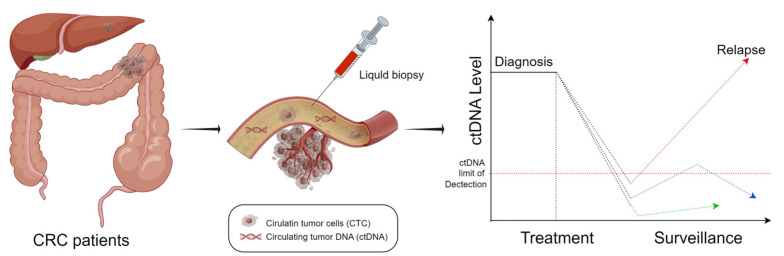
ctDNA during cancer progression. Created with: “Figdraw.com (accessed on 4 October 2023)”.

This article presents several studies on the application of MRD in clinical management of CRC, which identify and explore various aspects (more detailed subgroups of CRC patients, various MRD detection techniques). Despite the heterogeneity of the study designs, these data lead to a consistent conclusion: the identification of MRD through liquid biopsy, particularly ctDNA, can effectively predict the risk of early postoperative recurrence in CRC patients and to some extent guide ACT (Figure 1). It must be acknowledged that the introduction of the MRD concept offers an exceptionally effective tool to assess tumor recurrence. In the current context, the key aspect of MRD clinical application is to precisely identify “cured” patients. Therefore, negative predictive value becomes the most crucial indicator in MRD clinical application, while sensitivity is crucial. MRD assessment further refines the granularity of patient prognosis evaluation, providing a more effective tool for assessing the risk of recurrence in patients who have undergone curative surgery for CRC. We anticipate further advancements in ctDNA-based MRD techniques in the future, which may significantly enhance the precision of personalized targeted therapies for patients with mCRC.

## Figures and Tables

**Table 1:** Representative publications for clinical relevance of MRD at different stages of CRC.

Author	N	Stage	Risk of Recurrence in MRD+	Risk of Recurrence in MRD-	Evidence of ctDNA for MRD Techniques
Taieb et al. [39]	1017	III	31.4%	17.2%	ctDNA as an independent prognostic marker for DFS (adjusted HR = 1.55, 95% CI 1.13–2.12, *p* = 0.006) and OS (HR = 1.65, 95% CI 1.12–2.43, *p* = 0.011)
Benhaim, L. et al. [40]	184	II–III	44.4%	10.4%	ctDNA as a recurrence risk factor in stage II and III CRC before surgery and as a marker of MRD after surgery
Chen G. et al. [41]	240	II–III	60%	NA	ctDNA to guide decision-making in postsurgical management
Reinert T. et al. [42]	94	I–III	70%	11.9%	ctDNA enables risk stratification, ACT monitoring, and early relapse detection.
Wang Y. et al. [15]	58	I–III	100%	0%	ctDNA to stratify patients with resected CRC on the basis of risk of disease recurrence
Schøler LV. et al. [43]	27	I–III	100%	0%	Postoperative ctDNA detection provides evidence of MRD
Tie J. et al. [44]	178	II	78.6%	9.8%	ctDNA provides evidence of MRD in stage II colon cancer
Reinert T. et al. [45]	11	I–IV	100%	0%	ctDNA is a non-invasive, exquisitely specific and highly sensitive approach for monitoring disease load.
Henriksen TV. et al. [46]	160	III	96%	3%	The novel combination of ctDNA detection and growth rate assessment provides a strong prognostic value and enables tumor growth rate assessment.

MRD: minimal residual disease; CRC: colorectal carcinoma; ctDNA: circulating tumor DNA; DFS: disease free survival; HR: hazard ratio; CI: confidence interval; OS: overall survival.

## Data Availability

Not applicable.
